# Comparison of Visual and Quantra Software Mammographic Density Assessment According to BI-RADS^®^ in 2D and 3D Images

**DOI:** 10.3390/jimaging10090238

**Published:** 2024-09-23

**Authors:** Francesca Morciano, Cristina Marcazzan, Rossella Rella, Oscar Tommasini, Marco Conti, Paolo Belli, Andrea Spagnolo, Andrea Quaglia, Stefano Tambalo, Andreea Georgiana Trisca, Claudia Rossati, Francesca Fornasa, Giovanna Romanucci

**Affiliations:** 1 Private Practice, 44123 Ferrara, Italy; 2 AULSS 9 Scaligera, Breast Unit, Department of Radiology, 37122 Verona, Italy; 3 ASL ROMA 3, Department of Radiology, 00125 Rome, Italy; 4 UOC di Radiologia Toracica e Cardiovascolare, Dipartimento di Diagnostica per Immagini, Radioterapia Oncologica ed Ematologia, Fondazione Policlinico Universitario Agostino Gemelli IRCCS, 00168 Rome, Italy; 5 Facoltà di Medicina e Chirurgia, Università Cattolica Sacro Cuore, Largo F. Vito 1, 00168 Rome, Italy; 6 CIMEC-Center for Mind/Brain, University of Trento, 38068 Rovereto, Italy; 7 Private Practice, 37122 Verona, Italy

**Keywords:** mammographic density, automated software, breast imaging, screening, risk assessment, BI-RADS fifth edition, digital breast tomosynthesis

## Abstract

Mammographic density (MD) assessment is subject to inter- and intra-observer variability. An automated method, such as Quantra software, could be a useful tool for an objective and reproducible MD assessment. Our purpose was to evaluate the performance of Quantra software in assessing MD, according to BI-RADS^®^ Atlas Fifth Edition recommendations, verifying the degree of agreement with the gold standard, given by the consensus of two breast radiologists. A total of 5009 screening examinations were evaluated by two radiologists and analysed by Quantra software to assess MD. The agreement between the three assigned values was expressed as intraclass correlation coefficients (ICCs). The agreement between the software and the two readers (R1 and R2) was moderate with ICC values of 0.725 and 0.713, respectively. A better agreement was demonstrated between the software’s assessment and the average score of the values assigned by the two radiologists, with an index of 0.793, which reflects a good correlation. Quantra software appears a promising tool in supporting radiologists in the MD assessment and could be part of a personalised screening protocol soon. However, some fine-tuning is needed to improve its accuracy, reduce its tendency to overestimate, and ensure it excludes high-density structures from its assessment.

## 1. Introduction

Mammographic density (MD) refers to the changes in the radiological appearance of breast parenchyma linked to the variability in its composition, specifically in the amount of fatty and fibroglandular tissues. MD represents a double risk factor: firstly, high MD promotes the onset of certain types of breast cancer (BC) and, secondly, it produces a masking effect on screening mammograms that can hinder an early diagnosis of the disease [1].

It has been estimated that women with very dense breasts present a higher risk of developing BC of 4–6 times those with low BD [2,3,4,5,6]. The parenchyma components vary throughout a woman’s life, especially in the postmenopausal period, in which there is an involution of fibroglandular tissue [7]. Several studies showed that a great number of women when screened have an MD that belongs to higher categories [7,8].

It is, therefore, necessary that MD assessment is accurate and reproducible over time as this would enable clinicians to tailor the screening process to suit the patient’s needs and to add, if appropriate, complementary screening tests such as ultrasound (US) [9], magnetic resonance imaging (MRI) [10], or digital breast tomosynthesis (DBT) [11] (when not already integrated into the screening test) to improve the evaluation of the dense breasts and the detection of cancer. In this regard, it is emblematic that the United States affirmed the importance of this assessment with the introduction of breast density notification laws, by which physicians are required to inform patients about their MD and its implications.

In addition, breast density might be a surrogate indicator of treatment response: a decrease in MD after tamoxifen therapy is linked to a lower risk of primary BC, recurrence, and mortality [12,13,14].

Various methods based on qualitative, semi-automated, or fully automated approaches are available to measure MD on mammograms [15].

The first approach is mainly based on the American College of Radiology’s Breast Imaging Reporting and Data System (BI-RADS) Fifth Edition guidelines. These recommendations are routinely used by breast radiologists, who conduct a visual assessment to assign a density category.

According to the BI-RADS guidelines, MD is classified into four groups based on the relative amounts of adipose versus fibroglandular tissue, which are: (a) almost entirely fatty, (b) scattered fibroglandular density, (c) heterogeneously dense, and (d) extremely dense [16].

In semi-automated methods, the radiologist outlines the dense area, and a computer program calculates the MD.

Regarding the last approach, there are two main types of fully automated techniques: those that measure the percentage of MD based on the area (i.e., MedDensity and Autodensity) and those that perform a volumetric assessment of breast density, such as QuantraTM (Hologic Inc., Bedford, MA, USA) and VolparaTM (Volpara Solutions, Mãkatina Company, Wellington, New Zealand) [15]. Specifically, Quantra is based on a calculation algorithm, powered by artificial intelligence, that allows for a volumetric estimate of MD as a function of the density of the fibroglandular tissue and its distribution [17,18,19].

In all of the above, it has been demonstrated that MD is a risk factor for BC [5,20].

Nevertheless, visual assessment is affected by a subjective component in the interpretation, which implicates a significant variability between different readers (inter-observer variability) and with respect to the same reader over time (intra-observer variability) that could result in a discrepancy in the MD categorization [21,22,23]. The reader’s ability to accurately assess MD is probably influenced by two key factors: their experience and their ongoing commitment to professional development. A study has shown that readers who are actively engaged in continuing education and specialise in breast imaging exhibit minimal to no differences in their measurements, both in terms of consistency inter- and intra-observer, having instead only a moderate concordance with the software [16]. Conversely, another study pointed out that the greatest inter-observer agreement was among radiologists with the longest experience in breast imaging, underlining its importance, and, also, that the greatest disagreement was on the assignment of the most dense categories (C and D) [24].

Also, semi-automated techniques involve user interaction that still introduces subjectivity besides requiring additional complex software, further burdening the already overloaded radiology reporting workflow [25].

Moreover, knowledge and awareness of MD positively affect women’s approach to screening programs: women who are informed for the first time about their MD category intend to continue screening and conduct the necessary complementary examinations. Additionally, even women who have been informed several times about their MD continue to prioritise regular screening and healthy habits [26].

The points mentioned above underscore the necessity for standardised MD assessment, thereby enabling its inclusion as a parameter in clinical decision-making. It is precisely for this purpose that fully automatic systems have been devised.

This paper aims to evaluate the performance of breast density calculation software, verifying the degree of agreement with the gold standard, given by the consensus of two screening readers.

To this end, the density classes assigned by the two breast radiologists and the automatic software will be considered.

## 2. Materials and Methods

### 2.1. Study Population and Data Collection

The examinations were collected from women aged 50–74 years during the screening setting. A total of 5009 exams performed from February to April 2022 in two departments of UOSD Breast Unit ULSS 9 Scaligera, Ospedale Fracastoro in San Bonifacio and Ospedale di Marzana, were revised and included in this study. Participating women gave written informed consent.

The average age of the patients included in the study was 61 years (SD 7.3). The average age of patients with non-dense breast classification “A/B” was around 62 years (specifically, 61.6 years for category “A” and 62.6 years for category “B”) and with dense breast classification “C/D” was around 60 years (specifically, 60.6 years for category “C” and 57.2 years for category “D”).

Three MD assessments were assigned to these screening tests: two by breast radiologists, with 3 and 10 years of experience, respectively, and one by Quantra software. MD assessment data were collected in three groups, referred to as follows: Reader 1, Reader 2, and Software.

### 2.2. Image Acquisition

The examinations were acquired with a Selenia Dimensions Hologic mammography system and were composed of digital breast tomosynthesis acquisitions combined with two-dimensional synthetic mammography (2DSM). This system sent the raw images to the software server and the processed images to PACS for radiologists’ reporting. The server transmitted the images to the algorithm, which analysed them and returned the results in .xml format to the software’s server. Finally, the software’s server generated the results in DICOM format.

### 2.3. Software for Automatic Breast Density Assessment

Quantra™ 2.2.3 breast density assessment software (Hologic Inc.) was used for the automatic analysis of the images.

**Figure 1:**
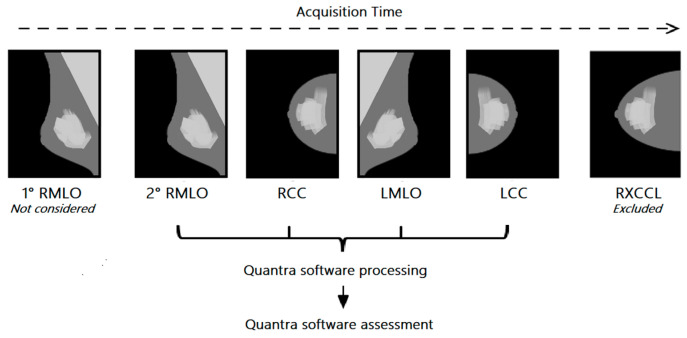
Raw images for MD assessment by Quantra Software.

This software analysed synthetic 2D and 3D images in 4 projections in DICOM format: right cranio-caudal, oblique mid-lateral, left cranio-caudal, and oblique mid-lateral. If the examination included multiple identical projections, e.g., two right cranio-caudal, just the second projection was considered for the assessment (Figure 1). Projections not recognized by the software were excluded.

Quantra 2.2.3 software produces an estimate of the MD based on the distribution and density of the parenchymal tissue using calculation algorithms provided by artificial intelligence.

It objectifies the MD assessment according to BI-RADS Fifth Edition recommendations. The system provides real-time visualisation of the assessment on the acquisition monitor, alongside the processed images, enabling customised diagnostic protocols.

Quantra analyses pixel data to quantify the characteristics of density and distribution of fibroglandular tissue. The characteristics are based on the variation in pixel values within the breast. The algorithm evaluates the density through a volumetric calculation of the amount of fibroglandular tissue present in each pixel. First, the software produces an estimate of the thickness of glandular tissue for each pixel in the image and calculates the total volume of fibroglandular tissue in the breast. Using a similar process, Quantra considers the contour of the breast on the image to compensate for unevenly compressed breast regions and to calculate the entire breast volume. The volumetric percentage of fibroglandular tissue is given by the ratio [27]:

$$Volumetricdensity=\frac{{Volume}_{fibro-ghiandulartissue}}{{Volume}_{wholebreast}}$$

Following BI-RADS Fifth Edition guidelines, the software evaluates the distribution of the fibroglandular tissue present. Subsequently, this information is used by a classification scheme to assess the density category of each breast.

**Figure 2:**
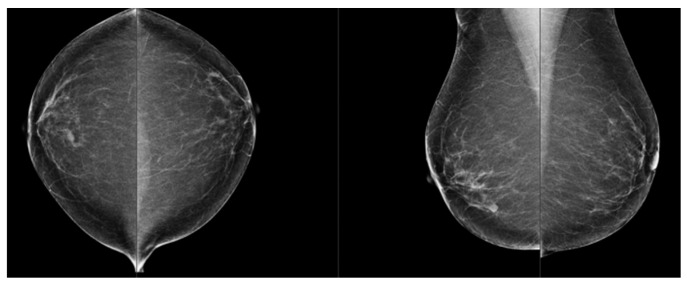
RCC, LCC, RMLO, and LMLO 2D-synthetic mammograms assigned to category A, “almost entirely fat”, by the Quantra 2.2.3 software according to BI-RADS lexicon.

**Figure 3:**
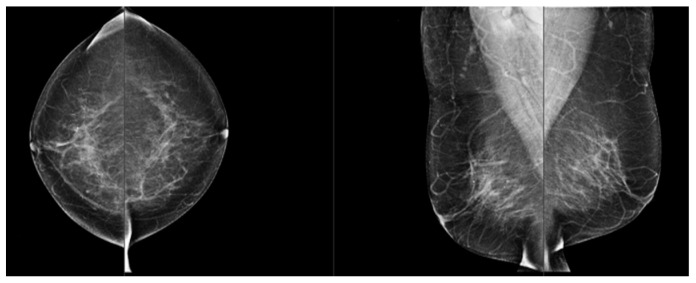
RCC, LCC, RMLO, and LMLO 2D-synthetic mammograms assigned to category B, “scattered fibroglandular”, by the Quantra 2.2.3 software according to BI-RADS lexicon.

**Figure 4:**
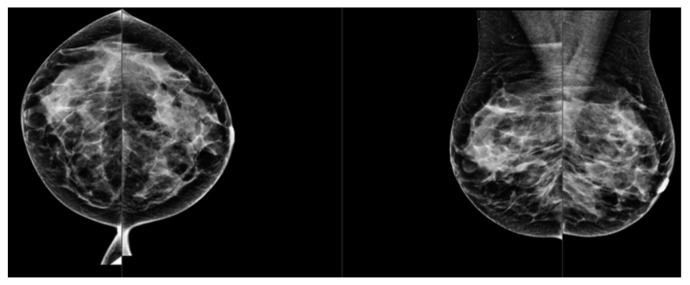
RCC, LCC, RMLO, and LMLO 2D-synthetic mammograms assigned to category C, “heterogeneously dense”, by the Quantra 2.2.3 software according to BI-RADS lexicon.

**Figure 5:**
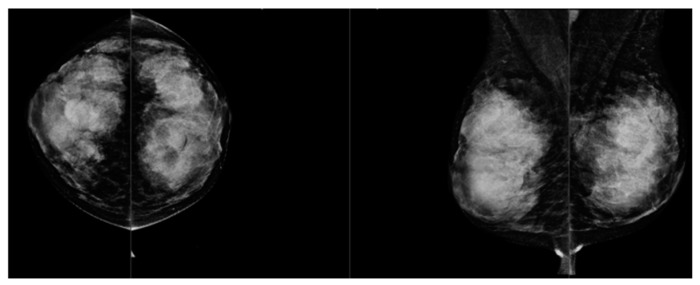
RCC, LCC, RMLO, and LMLO 2D-synthetic mammograms assigned to category D, “extremely dense”, by the Quantra 2.2.3 software according to BI-RADS lexicon.

The classification model is programmed using images previously classified by experienced radiologists to recognize densities correctly (Figure 2, Figure 3, Figure 4 and Figure 5).

**Figure 6:**
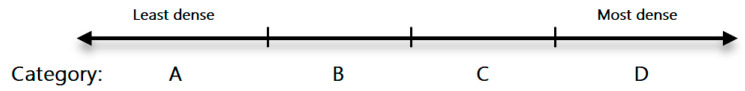
Density categories assigned by Quantra Software according to BI-RADS lexicon, fifth edition.

Quantra assigns density categories A, B, C, or D (Figure 6) to each breast and gives an estimate of breast density based on the distribution of fibroglandular tissue.

The final classification is given by the densest breast: for example, if the software has assigned a “B” to the right breast and a “C” to the left one, the final result would be a “C”.

### 2.4. Intraclass Correlation Coefficients

An analysis of the values assigned by breast radiologists and Quantra was conducted to extract the intraclass correlation coefficients (ICCs), which compare the different groups.

ICC represents a reliability index that indicates both correlation and agreement/reproducibility’ degree between measurements. It can be expressed as a value ranging between 0 and 1, where 1 represents a perfect match between measurements, and the different subranges suggest various levels of reliability [28]:

<0.50: poor correlation;

0.50–0.75: moderate correlation;

0.75–0.90: good correlation;

>0.90: excellent correlation.

## 3. Results

Quantra software is intended as a useful tool in supporting breast radiologists in the assessment of breast density, by eliminating humans’ subjective interpretation errors.

**Chart 1:**
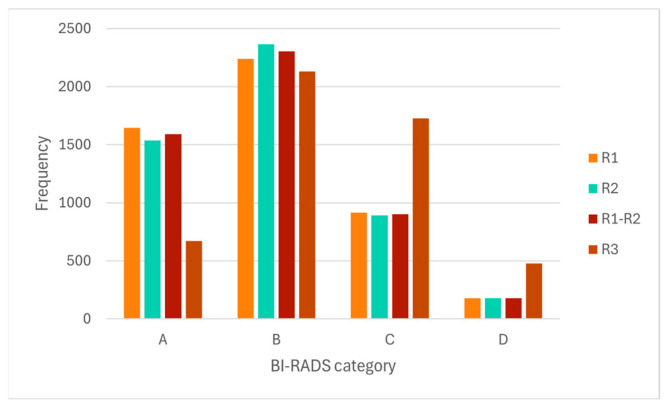
Graphic illustration of the data reported in Table 1 on the frequency of breast density categories assigned by each reader (R1 and R2), the mean of the two readers (R1-R2), and the Quantra software (R3).

The MD values assigned by Reader 1 (R1), Reader 2 (R2), the mean of the two readers (R1-R2), and the Quantra Software (R3) are reported in Table 1 and Chart 1.

Firstly, a comparison between the density assessment of reader 1 (R1) and the Quantra software (R3) for the same mammographic images was made and, subsequently, the correlation index was calculated. The resulting ICC was 0.725, which represents a moderate correlation between measurements. The same method was used to compare the assessment between reader 2 (R2) and the software, and, similarly, there was a moderate correlation with an index of 0.713. The correlation was higher when the comparison was made between the assessment of the software and the consensus of the two readers, indeed, the obtained index was 0.793, which reflects a good correlation. Nevertheless, some differences have also been observed in the visual assessment of the two radiologists: the ICC was 0.712, reflecting a moderate correlation. The ICC for each comparison is reported in Table 2.

As reported in Table 1 and Table 2, when considering the “B” category of breast composition (scattered fibroglandular density), there was a high correspondence between the Quantra software and human readers, as confirmed by the ICC. Conversely, the software assigned the non-dense “A” category (almost entirely fatty) to considerably fewer cases than the radiologists. This trend was reversed in the denser categories, i.e., “C” (heterogeneously dense) and “D” (extremely dense), with Quantra having assigned them more than the human readers.

**Figure 7:**
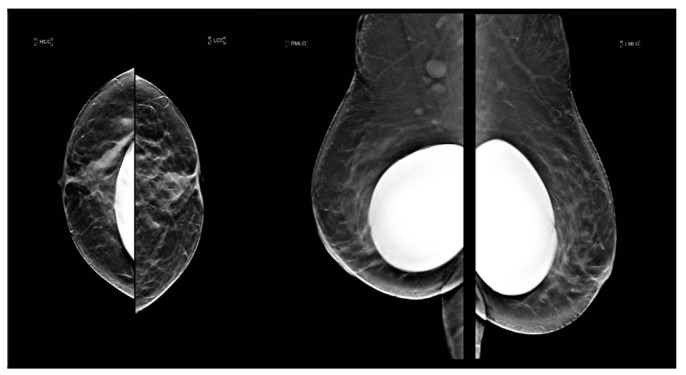
RCC, LCC, RMLO, and LMLO 2D-synthetic mammograms of the same patient. The visual assessment made by radiologists for this exam was “B”. Quantra software assigned the highest category, “D”, due to the presence of breast implants, resulting in an incorrect assessment.

**Figure 8:**
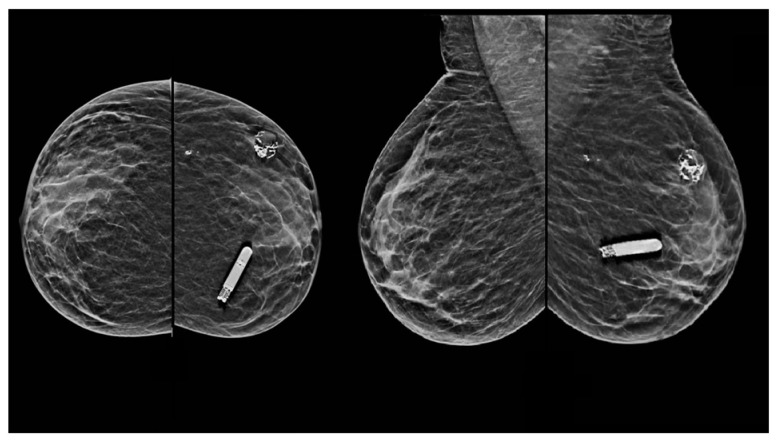
RCC, LCC, RMLO, and LMLO 2D-synthetic mammograms of the same patient. The visual assessment made by radiologists for this exam was “B”. Quantra software assigned the highest category, “D”, due to the presence of a loop recorder and some macrocalcifications in the left breast, resulting in an incorrect assessment.

To elucidate the discrepancies in density assessment between the radiologists and software for the assignment of the “D” category, a thorough review of the relevant cases was conducted. The analysis revealed that, in some instances, the discrepancy was attributed to the presence of high-density structures, such as calcifications or implantable devices (e.g., breast implants, pacemakers), overlapped with the breast parenchyma, that the software was unable to accurately recognize. In these cases, these structures acquired a greater value than the breast tissue in Quantra’s calculation formula, and the resulting assigned category was erroneously the highest, i.e., “D” (Figure 7 and Figure 8).

## 4. Discussion and Conclusions

An artificial intelligence tool, such as Quantra software, could be a useful tool for an objective and reproducible density assessment compared with the visual assessment provided by human readers, as it is not linked to experience or updating skills.

One potential drawback of introducing this software in clinical practice concerns the possibility that it can give a different assessment to human readers, potentially leading to overtesting and, therefore, higher healthcare costs, and probably, limited insurance coverage in some countries. Nevertheless, our results are promising. In fact, this study showed that the Quantra 2.2.3 software has a moderate agreement with individual readers and a good agreement with the consensus of two breast radiologists, as can be inferred from the intraclass correlation indices, on the analysis of 5009 examinations performed during the screening.

To the best of our knowledge, most of the studies in the literature have investigated software from different manufacturers or different versions of one software, older versions of the BI-RADS system for MD classification, or different statistical analysis methods, making it difficult to compare their findings to our results directly. For instance, in 2012, Ciatto et al. studied version 1.3 of Quantra software, suggesting the value of ≤22% as cut off to best reproduce visual classification, represented by a reading panel of 11 radiologists, in discriminating dense and non-dense breasts [29]. Similarly, in 2015, Pahwa et al. reported a cut-off of 19.5% for the same purpose [30]. A study was conducted to compare radiologists and Quantra 2.2 software assessment. This research was part of the evidence submitted to the FDA for approval of the software in the United States and the results showed that the overall agreement in the analysis of tomosynthesis images was 72%, lower than for conventional 2D images, but in the range of inter-reader variability [31]. In another published study, the reported agreement between human readers was substantial to almost perfect, whilst between the readers or the consensus of the readers and Quantra software was found to be moderate to substantial [32].

Additionally, our study has evidenced that the correspondence between the visual assessment and Quantra software assessment is greater in some categories rather than in others: in fact, the greater concordance was only for category “B”, while the software tends to overestimate the “dense” classes “C” and “D” when compared with radiologists.

Therefore, in a personalised screening setting in which patients require additional workup with ultrasound if the software automatically assigns a “D”, this overestimation could lead to an increased number of unnecessary recalls, raising concerns about its sustainability in clinical practice.

We encountered some limitations in this study. During the exams’ revision, we observed some errors in the assessment; in particular, the software was affected by the presence of high-density structures such as calcifications, breast implants, pacemakers, and other devices overlapped with the breast parenchyma. Indeed, these structures attenuate X-rays significantly more than the breast tissue. The algorithm was unable to distinguish between the different densities and their distribution. For this reason, if these structures were present, the software assigned the highest density, “D”. Consequently, we hope that before its introduction into clinical practice, Quantra can be updated to recognize and exclude such structures from the assessment. Alternatively, the presence of these structures in the breast parenchyma should be an exclusion criterion from software analysis.

Furthermore, ICC takes into account the correlation between multiple measures and, also, expresses a degree of reliability of the measurement, which indicates the level of confidence in the replicability of the measurement itself [28]. However, in the present dataset, given the subjective nature of the classification, which is grounded on human assessment, the rating may be misleading as potentially biased by misclassification. In this sense, computing the distance proposed assumes a certain degree of confidence in a ground-truth classification, which is not possible to define by design. To have a better insight into the results here proposed, additional metrics should be considered and integrated into future research.

A further limitation found during data collection was the lack of some density assessments by radiologists; nevertheless, as these data gaps were limited, their weight was negligible.

Finally, this was a monocentric study: further research in other radiology departments would be useful to confirm the accuracy of our data.

Overall, Quantra software appears to be a promising tool that could be part of a personalised screening protocol in the near future. However, before we can fully rely on it, it needs some fine-tuning. Specifically, its accuracy needs to be improved, its tendency to overestimate needs to be reduced, and its ability to correctly identify and exclude high-density structures from the assessment needs to be ensured.

## Figures and Tables

**Table 1:** The frequency of breast density categories assigned by each reader, the mean of the two readers, and Quantra software.

BI-RADS Category:	Reader 1 (R1)	Reader 2 (R2)	Mean R1-R2	Quantra Software (R3)
A	1646 (33%)	1538 (30%)	1592 (32%)	673 (13%)
B	2241 (45%)	2364 (48%)	2303 (46%)	2131 (43%)
C	915 (18%)	891 (18%)	903 (18%)	1728 (34%)
D	178 (4%)	179 (4%)	179 (4%)	477 (10%)

**Table 2:** Intraclass Correlation Coefficients.

Intraclass Correlation Coefficients	Results
ICC (R1 vs. R3)	0.725
ICC (R2 vs. R3)	0.713
ICC (R1 vs. R2)	0.712
ICC (meanR1-R2 vs. R3)	0.793

## Data Availability

The original contributions presented in the study are included in the article, further inquiries can be directed to the corresponding author/s.
